# The complete chloroplast genome of *Desmodium uncinatum* (Fabaceae)

**DOI:** 10.1080/23802359.2020.1852896

**Published:** 2021-03-11

**Authors:** Dengxia Yi, Wenbo Jiang, Lin Ma, Yongzhen Pang

**Affiliations:** Institute of Animal Sciences, Chinese Academy of Agricultural Sciences, Beijing, China

**Keywords:** Chloroplast genome, *Desmodium uncinatum*, Fabaceae

## Abstract

*Desmodium uncinatum* is one of the most important legume forage which distributes in tropical and subtropical regions of the world. In our study, we obtained the complete chloroplast genome of *D. uncinatum* with a length of 148,853 bp, including a large single copy region of 84,019 bp, small single copy region of 18,223 bp, and a pair of inverted repeat regions of 20,672 bp. The GC content in the whole chloroplast genome of *D. uncinatum* is 35.16%. Among the 133 unique genes in the circular genome, 37 tRNA, 12 rRNA and 84 protein-coding genes were successfully annotated. We constructed the Maximum likelihood (ML) tree with 11 species, and came to the conclusion that *D. uncinatum* was phylogenetically closely related to the genus of *Glycine* and *Trifolium*.

*Desmodium uncinatum*, important perennial forage legume native to Argentina, Bolivia, Brazil, Peru, and Venezuela, provides forage of high protein content to livestock (Ojija et al. 2019). In addition, *D. uncinatum* is used to control some problematic weeds and enhance crop production (Midega et al. 2017). *Desmodium uncinatum* is also widely used in traditional medicine for the treatment of neurological imbalances (Tsafack et al. 2018). The chloroplast is an important organelle that has its own genomes, and the chloroplast genome of plants has been a focus of research in plant molecular evolution and systematics (Clegg et al. 1994). Several features of chloroplast genome have facilitated molecular evolutionary analyses. However, the chloroplast genome sequences of *D. uncinatum* have not been reported so far. In the present study, chloroplast genes of *D. uncinatum* were sequenced and its structure features were characterized, which is a valuable resource for further studies of the Fabaceae family especially in terms of genetic evolution of forage crops.

Seeds of *D. uncinatum* was kept at the Forage Germplasm Bank at Institute of Animal Sciences of the Chinese Academy of Agricultural Sciences (Beijing, E116°29′, N40°03′). The voucher specimen (FR001) were deposited at the Herbarium of the Institute of Animal Sciences of the Chinese Academy of Agricultural Sciences, Beijing, China. After germinate in lab, genomic DNA from young leaves was extracted using a DNA Extraction Kit from Tiangen Bio Tech Co., Ltd (Beijing, China). The sequencing was carried out on the Illumina Novaseq PE150 platform (Illumina Inc, San Diego), and 150 bp paired-end reads were generated. The software GetOrganelle v1.5 (Jin et al. 2018) was used to assemble the cleaned reads into a complete chloroplast genome, with the chloroplast genome of *Medicago truncatula* (GenBank accession number: NC_003119) as a reference. The chloroplast genome annotation was performed through the online program CPGAVAS2 (Shi et al. 2019) and GeSeq (Tillich et al. 2017), followed by manual correction. The assembled chloroplast genome sequence has been submitted to GenBank with the accession number MT528595.

In the present study, the complete chloroplast genome of *D. uncinatum* is 148,853 bp in length, which is a typical circular structure, consisting of two reverse repeat regions (IRa and IRb) of 20,672 bp that were separated by a large single copy (LSC, 84,019 bp) and a small single copy (SSC, 18,223 bp). The GC content of the whole chloroplast genome is 35.16%. The chloroplast genome is identified to have genes of 133 in total, including 84 protein-coding genes, 37 tRNA genes, and 12 rRNA genes. Among them, 36 genes encoding amino acid transfer protein (trnP-UGG, trnW-CCA, trnQ-UUG, trnS-GCU, trnR-UCU, trnC-GCA, trnE-UUC, trnY-GUA, trnD-GUC, trnT-GGU, trnS-UGA, trnG-GCC, trnM-CAU, trnS-GGA, trnT-UGU, trnL-UAA, trnF-GAA, trnV-UAC, trnM-CAU, trnK-UUU, trnH-GUG, trnL-CAA, trnV-GAC, trnE-UUC, trnA-UGC, trnR-ACG, trnN-GUU, trnL-UAG, trnN-GUU, trnR-ACG, trnA-UGC, trnE-UUC, trnV-GAC, trnL-CAA, trnM-CAU, and trnM-CAU*-*), 25 genes encoding ribosomal structural proteins protein (rps19, rpl22, rps3, rpl16, rpl14, rps8, rpl36, rps11, rps12, rpl20, rps18, rpl33, rps16, rps2, rps14, rps4, rps19-fragment, rpl2, rpl23, rps7, rpl32, rps15, rps7, rpl23, and rpl2 ), 16 genes encoding electron transport protein (petD, petB, petG, petA, petN, ndhJ, ndhK, ndhC, ndhB, ndhF, ndhD, ndhG, ndhI, ndhA, ndhH, and ndhB), 14 genes encoding light collection structural protein (PSII) (psbH, psbT, psbB, psbE, psbF, psbL, psbJ, psbK, psbI, psbM, psbD, psbC, psbZ, and psbA) are found in the chloroplast genome of *D. uncinatum* (Figure 1).

**Figure 1.**
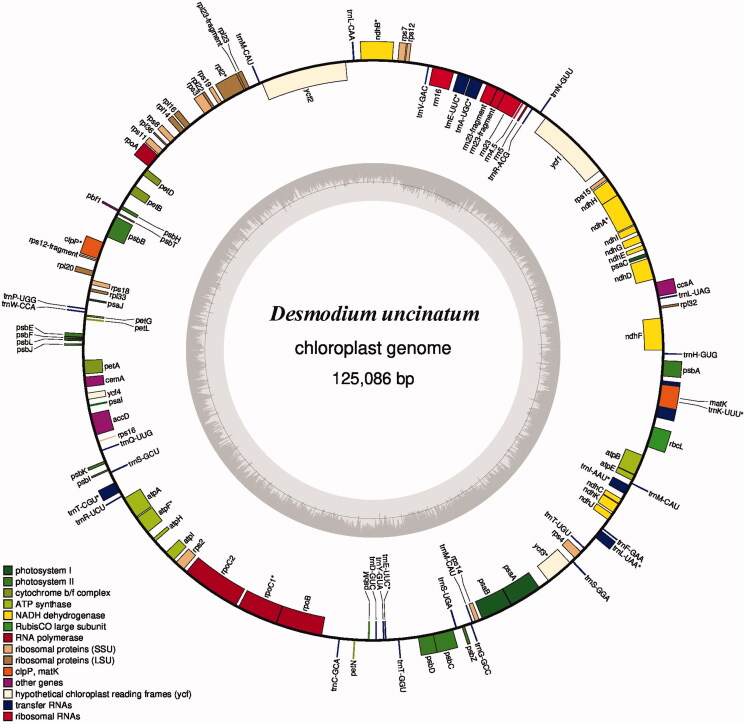
Gene map of the *D. uncinatum* chloroplast genome. The genes shown inside of the circle indicates transcriptional direction is clockwise, while those shown outside are counterclockwise. Genes belonging to different functional groups are indicated in different colors. The GC content of the genome shown with gray histogram in the inner circle, and the gray line depicts the 50% threshold line.

The chloroplast genomes of 10 species from Fabaceae and as well as *Vachellia flava* as outgroup species was downloaded from the NCBI GenBank database to identify the phylogenetic relationship of *D. uncinatum*. The sequences were aligned using MAFFT v7 (Katoh et al. 2019). In addition, a Maximum likelihood (ML) tree based on the common protein-coding genes of 11 species was constructed by using raxmlGUI1.5b (v8.2.10) (Silvestro and Michalak 2012). Phylogenetic analysis shows that *Trifolium meduseum* and *Desmodium renifolium* are closely related to *D. uncinatum* (Figure 2). This study will provide important information for species identification, and phylogenetic relationship in Fabaceae family, in particular for legume forage.

**Figure 2.**
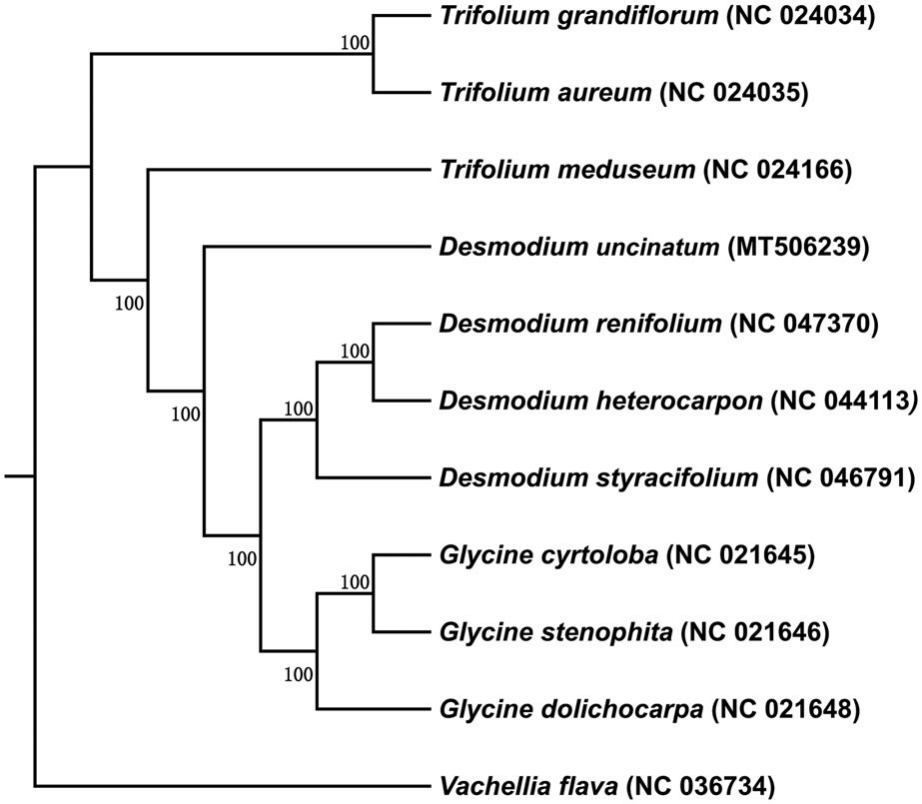
Phylogenetic tree reconstructed using maximum likelihood (ML) method based on the common protein-coding genes of 10 species of the Fabaceae family, with V. flava as the outgroup. Numbers above the lines represent ML bootstrap values (>70%).

## Data Availability

The data that support the findings of this study are openly available in NCBI at Genbank with accession number MT528595 (https://www.ncbi.nlm.nih.gov/nuccore/MT528595). Raw sequencing reads used in this study was deposited in the public repository SRA with accession number SRR12744902 (https://www.ncbi.nlm.nih.gov/sra/?term=SRR12744902).
